# What Defines Early Specialization: A Systematic Review of Literature

**DOI:** 10.3389/fspor.2020.596229

**Published:** 2020-10-27

**Authors:** Alexandra Mosher, Jessica Fraser-Thomas, Joseph Baker

**Affiliations:** School of Kinesiology and Health Science, York University, Toronto, ON, Canada

**Keywords:** early specialization, definition, sampler, review, specializer

## Abstract

**Introduction:** While practitioners and organizations advise against early specialization, the lack of a consistent and clear definition of early specialization reduces the impact of recommendations and policies in youth sport. An important first step in understanding the consequences of early specialization is establishing what early specialization is.

**Objectives:** This PRISMA-guided systematic review aimed to determine the types, characteristics, and general content of early specialization papers within the literature, and examine how early specialization has been defined and measured in order to advance knowledge toward a clear and consistent definition of early specialization.

**Data sources:** Four different electronic databases were searched (SPORTDiscus, Web of Science, Sports Medicine and Education Index, and Scopus). Both non data-driven and data-driven studies were included to ensure a comprehensive understanding of the literature.

**Eligibility Criteria:** In order to be included in the review, the paper must: (a) Focus on specialization and explicitly use the term “specialization” (b) Focus on sport and athletes (c) Be papers from a peer-reviewed (d) Be in English. And finally, (e) be available in full text.

**Results:** One thousand three hundred and seventy one articles were screened resulting in 129 articles included in the review after applying inclusion/exclusion criteria. Results indicated a clear discrepancy between key components of early specialization and the approaches used to classify early specializers.

**Conclusion:** Future research should work toward developing a valid and reliable approach to classifying early specializers and establishing a consistent definition across studies.

## Introduction

In 2016, the Canadian Lifestyle and Fitness Research Institute reported 77% of youth aged 5–19 participated in organized physical activity or sport. According to the Aspen Institute's Project Play (The Aspen Institute Project Play, 2019), 38% of children aged 6–12 participated in sport on a regular basis in 2018, based on United States government population statistics (Federal Interagency Forum on Child Family Statistics, 2020), equating to ~9 million American children participating in sport regularly. Similarly, Australia reported 72.3% of children under the age of 15 participated in some type of sport related activity in 2019 (May, 2019), while in England 86.4% of children ages 5–15 were reported to participate in sport in 2018 (Lange, 2019). Due to the large number of youths participating in sport globally, researchers have attempted to better understand common sport pathways, and the benefits or consequences of sport participation.

One element of youth sport that has received more attention in recent years is early specialization, originally posited as athletes focusing on one sport that is practiced, trained and competed in year-round (Hill, 1993). Models of athlete development (e.g., Developmental Model of Sport Participation) (Côté and Fraser-Thomas, 2016) suggest early specialization excludes an important period of development where youth should be participating in a range of sports with the purpose of fun and enjoyment, in favor of dedication and skill acquisition in one sport. However, expertise-centered models of skill development (e.g., Deliberate Practice Framework) (Ericsson et al., 1993) suggest that individuals who begin focused practice early have an advantage over those who start later. Despite the prominence of the notion of deliberate practice in discussions of coaching and athlete development, a growing body of literature suggests early specialization is not a prerequisite of becoming an elite athlete (Soberlak and Cote, 2003; Buckley et al., 2017; Huxley et al., 2017; Black et al., 2019). Further, particular indicators of early specialization have been linked to a host of negative consequences. Researchers have found those who specialize early are at greater risk of injury, experience increased exhaustion, and are more likely to dropout than athletes who do not (Fraser-Thomas et al., 2008; Strachan et al., 2009; Bell et al., 2018c).

Over the past 20 years, at least seven major national and international sport and athletic associations, societies, federations, and organizations have released position statements advising against the practice of early specialization amongst youth athletes (e.g., American Orthopedic Society for Sports Medicine, American Academy of Pediatrics, International Society of Sport Psychology, National Association for Sports and Physical Education). Such strong consensus suggests there is clear and unambiguous evidence that early specialization is harmful and should be avoided in any context; however, further investigation indicates the evidence against early specialization may not be as robust as these position statements make it seem.

To begin, there are very few studies explicitly studying the consequences of early specialization; instead the literature is comprised heavily of review papers, commentaries, and editorials that reiterate previous findings. For example, a 2018 meta-analysis on specialization and overuse musculoskeletal injuries was comprised of only four studies (Bell et al., 2018c), suggesting an overall lack of research. More importantly, there is no standard definition of early specialization. Several researchers have emphasized the lack of a clear and consistent definition and have suggested that this inconsistency makes it unclear what exactly constitutes early specialization (Ferguson and Stern, 2014; Buckley et al., 2017). Some have defined early specialization as “year round intensive training in a single sport at the exclusion of other sports” (Jayanthi et al., 2015) while others proposed “the time when the athlete defined one sport as being more important than other sports” (Moseid et al., 2019). Further complicating conceptualizations, some have suggested it is the type of participation (i.e., deliberate practice) that is a key marker of early specialization (Hendry and Hodges, 2018) while others designate early start age and early involvement in competitive sport as key parameters of early specialization (Baker et al., 2009). Without a consistent definition of early specialization, it is difficult to conclude early specialization is as harmful to youth as many organizations are claiming. More importantly perhaps—the lack of a clear definition of this phenomenon makes improving developmental training environments difficult given it is not clear what element of specialization (e.g., intensity, early start age, over-emphasis on winning) may be driving any negative consequences that do exist.

A recent systematic review of early specialization (DiSanti and Erickson, 2019) found that only 13 of 40 studies operationally defined “specialization.” Among the few studies that provided an operational definition of specialization, the criteria used to distinguish early specializers varied considerably. Given these inconsistent criteria, athletes could be classified into different groups depending on the definitions used, raising concerning questions of reliability and validity of conclusions regarding early specialization. An important next step in determining the relationships between early specialization and developmental and performance outcomes, as well as identifying the mechanisms behind these effects, is to clearly define early specialization.

Practice and research in sport psychology is strongly influenced by policy decisions, and therefore, unlike previous reviews which have examined only data-driven studies (Fabricant et al., 2016; Bell et al., 2018c; Walters et al., 2018), this review will also include non-data driven articles. This will provide a more thorough understanding of the current state of literature (not just the state of the research) and overall understanding of the conceptions of “early specialization” in sport psychology and related fields of study. We believe this variation to the formula of systematic reviews makes this a novel approach to understanding a concept in its entirety.

It is important to note this review did not focus on scientific or measurement-related issues concerning definitions of early specialization (e.g., the implications of a yes/no dichotomy of specialization vs. a continuous measure). The necessary evidence for an empirically substantiated definition of early specialization has yet to be established and while these issues are clearly important in the study of early specialization, they were outside the scope of this review.

The aim of this review was not to come to a conclusion about the potential consequences or benefits of early specialization, as has been done in the past; the goal of this review was to gain a thorough understanding of the entire breadth of literature on the subject. As such, the objectives of this systematic review were: (a) to determine the types, characteristics, and general content of early specialization papers within the literature, and (b) to examine how early specialization has been defined and measured in the sport literature across all fields of study (e.g., biomechanics, psychology, talent development) and populations, in order to advance knowledge toward a clear and consistent definition of early specialization.

## Methods

### Research Protocol

The Preferred Reporting Items for Systematic Reviews and Meta-Analyses (PRISMA) statement (Moher et al., 2009) was used as a guide for the exploration of literature. There is no protocol registered for this review.

### Eligibility

In order to be included in the review, a priori criteria were established; specifically, the paper must: (a) Focus on specialization and explicitly use the term “specialization”; this meant that specialization had to be one of the key elements of the paper and not a footnote or added section. (b) Focus on sport and athletes; this ensured the focus was on sport specialization and not any other type of specialization (e.g., as it relates to medical expertise). (c) Be papers from a peer-reviewed journal rather than exclusively empirical studies; any review, commentary, editorial etc. was eligible for inclusion, in order to capture any and all definitions of early specialization and a more comprehensive picture of the current state of the literature^1^. (d) Be in English. And finally, (e) be available in full text.

### Information Sources and Search Strategy

Beginning in June 2019, in consultation with a professional research librarian a rigorous search strategy was created. To identify relevant literature, thoroughly thought out search strings and key words were used within four electronic databases (i.e., SPORTDiscus, Web of Science, Sports Medicine and Education Index, and Scopus). Key words included “specialize” and “sport” as well as synonyms such as “year-round training” or “single-sport.” For the keyword “early” synonyms included “youth,” “child,” and “adolescent.” Various combinations of these key words were used for each of the four databases. In order to ensure studies captured all components, the connector “AND” was used, and to capture all variations, truncation was used. An example of a search string used in the Scopus database is (specialize^*^ AND early AND sport^*^). In order to get a thorough understanding of the research into early specialization, papers could be published any time before June 2019, with a final search date of August 2019.

### Study Selection

**Figure 1:**
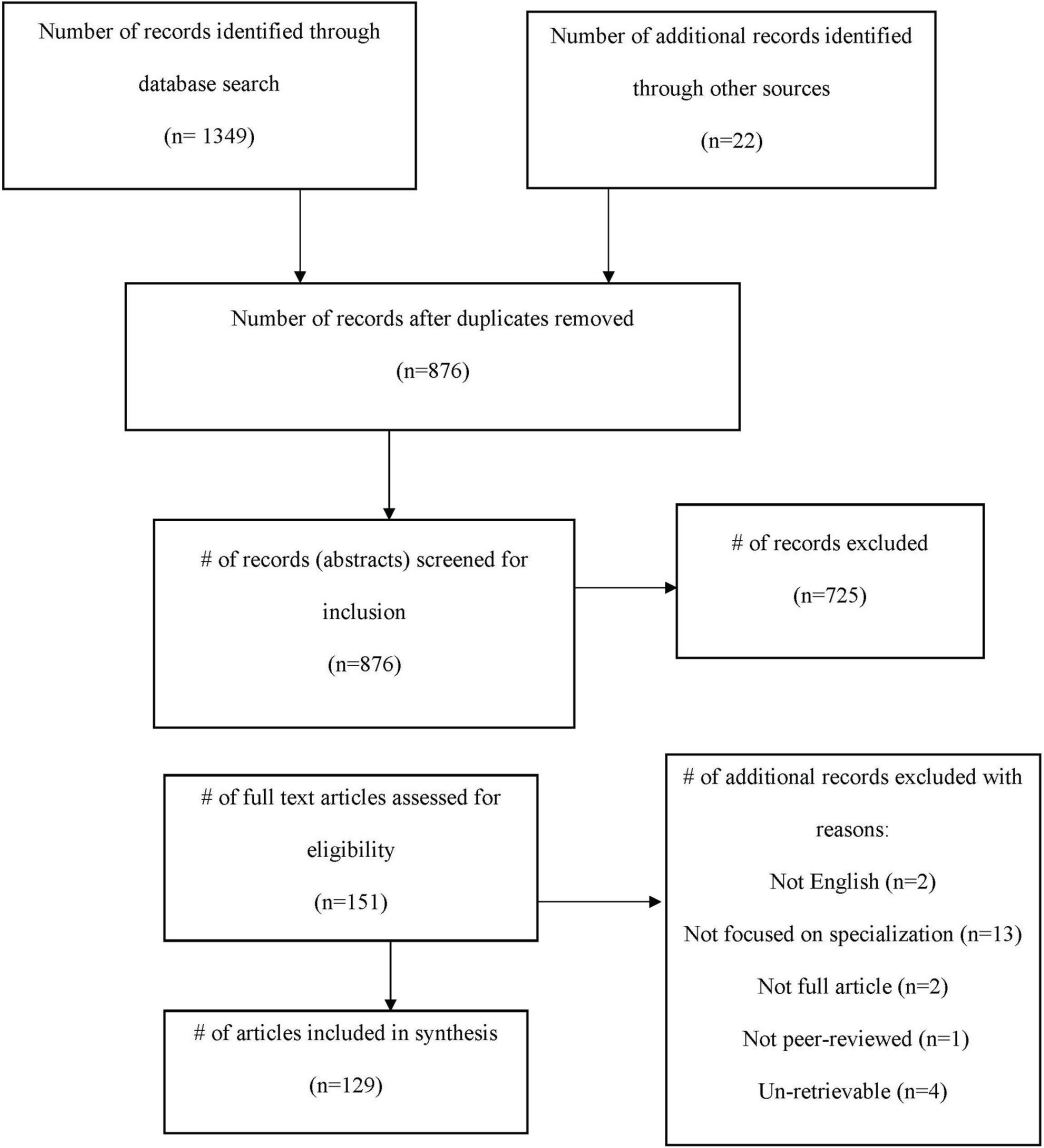
PRISMA flow chart outlining flow of information through the different phases of the systematic review.

The initial search resulted in 1,349 articles. An additional 22 were identified from reference lists of seminal papers, creating a total of 1,371. After duplicates were removed, 876 articles were screened. Information from all articles including title, year of publication, authors and abstract was compiled in an excel document for organization purposes. At this stage, the titles and abstracts for all articles were screened based on the above criteria, in order to determine inclusion or exclusion. If the first author was unsure, another author was consulted, and discussion continued until a decision was reached. This screening resulted in the exclusion of 725 articles, with 151 articles for full text review. Of the 151 articles read in-full, two were found to not be in English, 13 were deemed to have not focused on sport specialization, two were conference proceedings, one was not peer-reviewed and four were un-retrievable for a total of 22 studies being excluded in this step, resulting in a final total of 129 studies included in the systematic review. For a complete flow chart, see Figure 1.

### Data Collection

The remaining 129 articles were then put into a new spreadsheet for data extraction. The definition used for early specialization and purpose of each study were transferred to this new file to allow for further analysis. To cover the objectives of the review, for empirical studies, additional information regarding methods used, sample size, country of study, age and sex of sample as well as sport studied were extracted from each paper.

### Risk of Bias and Additional Analysis

Given that the objectives of this review were to determine the types, characteristics, and general content of early specialization papers within the literature, and to examine how early specialization has been defined and measured in the sport literature (i.e., not to summarize outcomes) a bias assessment was not performed. Additionally, because this was not a meta-analysis, no additional statistical analyses (e.g., meta-regression) were performed on the collected papers.

## Results

### Paper Types, Characteristics, and Content

To achieve the first objective of the review, studies were first categorized based on article type (i.e., non-data-driven editorials/commentaries/reviews, systematic reviews/meta analyses, and data driven studies), to gain a better understanding of the overall composition of the literature. Of the 129 papers included in the study, 43.4% (*n* = 56) were non-data driven papers (i.e., editorials, reviews and commentaries) and 3.8% (*n* = 5) were systematic reviews. The data-driven studies (*n* = 68; 52.7%) were further divided into those that explicitly included specialization in the purpose (i.e., specialization specific; *n* = 48; 37.2%) and those that did not include specialization in the purpose but met all criteria to be included in the review (i.e., specialization general; *n* = 20; 15.5%).

Table 1 provides an overview of the characteristics of non-data driven papers which included 36 reviews, eight commentaries, seven editorials, and five position/consensus statements. Among this category, the areas of focus were injury, talent development and policy (for full breakdown see Table 1). The five position statements were from five different organizations, which were all either against or relatively neutral toward early specialization, indicating a negatively skewed perception of early specialization.

**Table 1:** Characteristics of non-data driven papers.

**References**	**Type**	**Organization**	**Position**	**Area**
American Academy of Pediatrics (2000)	Position Statement	American Academy of pediatrics	Neutral	Physical
Anderson and Mayo (2015)	Review		Neutral	Skill Acquisition
Baker and Robertson-Wilson (2003)	Review		Neutral	Talent and development
Baker et al. (2009)	Review		Neutral	Multidisciplinary
Baker (2003)	Review		Neutral	Talent and development
Bell (2018)	Editorial		Neutral	Physical
Blagrove et al. (2017)	Review		Against	Physical
Bodey et al. (2013)	Review		Neutral	Multidisciplinary
Branta (2010)	Review		Neutral	Fundamental motor skills
Brenner (2016)	Position Statement	American Academy of Pediatrics	Against	Physical
Brylinsky (2010)	Review		Neutral	Coaching
Callender (2010)	Review		Neutral	Multidisciplinary
Capranica and Millard-Stafford (2011)	Commentary		Neutral	Physiological and talent
Carson et al. (2010)	Editorial		Neutral	Multidisciplinary
Coakley (2010)	Review		Against	Parenting
Côté and Hancock (2016)	Review		Neutral	Policy
Coté et al. (2009)	Review		Against	Psychosocial
Côté et al. (2009)	Position Statement	International Society of Sport Psychology	Against	Development
DiFiori et al. (2017)	Editorial		Against	Talent and sport participation
DiFiori et al. (2014)	Position Statement	American Medical Society for Sports Medicine	Against	Multidisciplinary
Feeley et al. (2016)	Review	American Orthopedic Society for Sports Medicine	Against	Physical
Geisler (2019)	Editorial		Neutral	Decision to specialize
Gonçalves et al. (2012)	Commentary		Neutral	Talent identification
Goodway and Robinson (2015)	Commentary		Against	Physical growth, motor development
Gould (2010)	Review		Neutral	Psychological
Griffin (2008)	Review		Against	Multidisciplinary
Hastie (2015)	Review		Neutral	Pedagogy
Haugaasen and Jordet (2012)	Review		neutral	Soccer talent
Horn (2015)	Review		Neutral	Multidisciplinary
Jayanthi and Dugas (2017)	Review		Against	Physical
Kaleth and Mikesky (2010)	Review		Neutral	Physiological
Landers et al. (2010)	Editorial		Neutral	Multidisciplinary
LaPrade et al. (2016)	Consensus statement	American Orthopedic Society for Sports Medicine	Against	Multidisciplinary
Malina (2010)	Review		Neutral	Multidisciplinary
Mattson and Richards (2010)	Review		Neutral	Biomechanical
Matzkin and Garvey (2019)	Review		Against	Physical
Mostafavifar et al. (2013)	Editorial		Against	Multidisciplinary
Myer et al. (2015)	Review		Against	Multidisciplinary
Myer et al. (2016)	Review		Against	Multidisciplinary
NASPE Staff (2006)	Commentary	National Association of Sport and Physical Education	Against	Multidisciplinary
Normand et al. (2017)	Review		Neutral	Physical and psychological development
Pantuosco-Hensch (2006)	Commentary		Neutral	Multidisciplinary
Read et al. (2016)	Review		Against	Physical
Reider (2017)	Editorial		Neutral	Multidisciplinary
Sluder et al. (2017)	Review		Neutral	Multidisciplinary
Smith (2015)	Review		Neutral	Historical
Smith et al. (2017)	Review		Neutral	Multidisciplinary
Smucny et al. (2015)	Review		Against	Multidisciplinary
Stewart and Shroyer (2015)	Commentary		Neutral	Multidisciplinary
Sugimoto et al. (2017)	Review		Against	Physical and Talent
Torres (2015)	Review		Neutral	Philosophical
Waldron et al. (2019)	Review		Neutral	Multidisciplinary
Weiss (2015)	Commentary		Neutral	Multidisciplinary
Wiersma (2000)	Review		Neutral	Multidisciplinary
Williams (2018)	Commentary		Neutral	Physical
Wilson (2006)	Review		Against	Multidisciplinary

Table 2 presents the data from the systematic reviews. The number of studies included in each review ranged from 3 to 40. Injury was the main focus of these reviews (*n* = 3) while the remaining two were multidisciplinary in nature.

**Table 2:** Characteristics of systematic reviews.

**References**	**Number of studies**	**Outcome**
	**included**	**studied**
Bell et al. (2018c)	5	Injury
DiSanti and Erickson (2019)	40	Multidisciplinary
Fabricant et al. (2016)	3	Injury
Jayanthi et al. (2013)	Did not specify	Multidisciplinary
Walters et al. (2018)	Did not specify	Injury and development

The characteristics of the data-driven studies are provided in Table 3. Within the 48 studies in the specialization specific category (i.e., explicitly included specialization in the purpose), there were a variety of outcomes studied. Injury studies (*n* = 14) were the most prominent and were often epidemiological examinations of rates or risk of injury in early specializers. Specialization characteristics such as age of specialization or prevalence were also heavily studied (*n* = 10). Talent development studies (*n* = 9) focused on the training activities of elite athletes, often comparing them to their less successful peers. Psychological outcomes (e.g., burnout, mental toughness) and physical outcomes (e.g., landing error, anterior y balance performance), were less heavily studied (i.e., *n* = 5 and *n* = 4, respectively). The least studied areas in relation to early specialization were later physical activity (*n* = 3), and skill transfer to other sports (*n* = 1). There were also single studies that considered how specialization affected (a) ability to learn basketball skills in non-basketball players (Santos et al., 2017), and (b) health related quality of life (Patel and Jayanthi, 2018). The average sample size of studies in this category was 499.7 with a range from 1 to 3090. The studies were comprised of retrospective (*n* = 16), cross-sectional (*n* = 15), case control (*n* = 8), descriptive epidemiological (*n* = 4), longitudinal (*n* = 1), prospective (*n* = 1), case study (*n* = 1), case report (*n* = 1), and a single cohort studies. Studies came from a total of 11 different countries and there was a large variety of individual and team sports examined.

**Table 3:** Summary of data-driven study characteristics.

**References**	**Sex of sample**	**Age of sample**	**Sample size**	**Country**	**Sport**	**Study design**	**Outcome(s) studied**
**Specialization Specific**							
Beese et al. (2015)	Female	Highschool	40	USA	Soccer	Cross-sectional	Landing error
Bell et al. (2016)	Male/Female	13–18	302	USA	Soccer, basketball, tennis, volleyball	Cross-sectional	Prevalence in high school
Bell et al. (2018b)	Male/Female	High school	354	USA	Volleyball, tennis, basketball, soccer	Cross-sectional	Specialization characteristics
Bell et al. (2018a)	Male/Female	12–18	761	USA	Soccer	Cross-sectional	Injury
Black et al. (2019)	Male	18–39	91	USA	Ice hockey	Retrospective	Age of specialization
Bridge and Toms (2013)	Male/Female	7–18	1,006	UK	Athletics, football, hockey, netball, rugby union, swimming, boxing, power lifting	Retrospective	Talent
Brooks et al. (2018)	Male/Female	12–18	974	USA	Baseball, basketball, cheer/dance, cross-counrty, football, gymnastics, ice hockey, lacrosse, soccer, softball, swimming, tennis, track, volleyball, wrestling	Cross-sectional	Knowledge attitudes and beliefs of specialization
Buckley et al. (2017)	Male/Female	14–26	3,090	USA	Did not specify	Retrospective	Rate and age of specialization, the number of months per year of single-sport training, and the athlete's perception of injury related to specialization.
Buhrow et al. (2017)	Male/Female	18–23	102	USA	Swimming/diving, golf, basketball, track and field/cross-country, softball, tennis, football, wrestling, soccer	Cross-sectional	Mental toughness
DePhillipo et al. (2018)	Male	12	11	USA	Alpine skiing	Case Report	Injury
DiCesare et al. (2019)	Female	Adolescent	79	USA	Basketball, Soccer, volley ball	Case control	Lower extremity biomechanical deficits
DiStefano et al. (2018)	Male/Female	8–15	355	USA	Soccer, basketball	Cross-sectional	Landing technique
Ferguson and Stern (2014)	Male	16	1	Canada	Baseball	Case Study	Injury
Ford et al. (2012)	Male/Female	Under 16	328	Brazil, England, France, Ghana, Mexico, Portugal Sweden	Soccer	Retrospective	Talent
Gallant et al. (2017)	Male/Female	10–11 at start	756	Canada	Did not specify	Longitudinal	Physical activity and participation patterns
Ginsburg et al. (2014)	Male	18–39	708	USA	Baseball	Retrospective	Talent
Hall et al. (2015)	Female	middle and high school	357	USA	Basketball, soccer, volleyball	Retrospective	Injury
Hill (1993)	Male	adults	152	USA	Baseball	Retrospective	Talent
Jayanthi et al. (2015)	Male/Female	7–18	1,190	USA	Did not specify	Clinical case control	Injury
Jayanthi et al. (2018)	Male/Female	7–18	1190	USA	Did not specify	Cohort study	Injury, SES
Larson et al. (2019)	Male/Female	12–13	137	Canada	Swimming	Retrospective	Psych, burnout and dropout
Martin et al. (2017)	Male/Female	College students	1,041	USA	Football, track and field, soccer, cross country, swimming, diving, baseball, wrestling, basketball, golf, tennis, rowing, gymnastics, volleyball, field, hockey, softball, figure skating	Retrospective	Prior sport experience, importance of specialization and Talent
McDonald et al. (2019)	Did not specify	University/Olympic	143	USA	wrestling	Descriptive epidemiological study	Injury
McFadden et al. (2016)	Male	13–18	61	Canada	Ice hockey	Case control	Psychological needs satisfaction, mental health
McGuine et al. (2017)	Male/Female	High school	1,544	USA	Baseball/softball, basketball, football, soccer, tennis, track/cross-country, volleyball, wrestling,	Prospective	Injury
McLeod et al. (2019)	Male/Female	12–18	746	USA	Soccer	Cross-sectional	Soccer participation and specialization characteristics
Mendes et al. (2018)	Male	Under 19 under 21	78	Brazil	Volleyball	Retrospective	Talent
Miller et al. (2017)	Male/Female	High school	295	USA	Basketball, soccer, volleyball, tennis	Cross-sectional	Anterior y balance performance, sex
Moseid et al. (2019)	Male/Female	16	259	Norway	Did not specify	Cross-sectional	Injury and illness
Noble and Chapman (2018)	Male	Adults	519		Marathon	Retrospective	Talent
Padaki et al. (2017a)	Male/Female	Youth	201	USA	Soccer, basketball, baseball/softball, lacrosse, cross-country/track and field, football, swimming, tennis	Cross-sectional	Parental influence
Padaki et al. (2017b)	Male/Female	7–18	235	USA	Soccer, basketball, baseball/softball, lacrosse, cross-country/track and field, football, hockey, volleyball, swimming, tennis, gymnastics	Descriptive epidemiological study	Factors for specializing
Pantuosco-Hensch (2010)	Male/Female	17–23	469	USA	Lacrosse, soccer, swimming, tennis	Retrospective	Perceptions of ES
Pasulka et al. (2017)	Male/Female	7–18	1190	USA	Soccer, basketball, volleyball, baseball/softball, football, cheer, hockey, lacrosse, badminton, tennis, gymnastics, dance, swimming, wrestling, track andfield, cross-country, martial arts, diving, figure skating, horseback riding, downhill skiing, fencing, golf	Clinical case control	Injury
Patel and Jayanthi (2018)	Male/Female	8–15	50 child 42 parents	USA	Tennis, gymnastics, soccer, basketball, swimming, football, golf, wrestling, track	Case control	Health related quality of life
Post et al. (2017c)	Male/Female	12–18	2011	USA	Soccer, basketball, swimming/diving, ice hockey, volleyball, track/cross-country, lacrosse, baseball, football, softball, cheer/dance, gymnastics, tennis, wrestling	Case control study	Injury
Post et al. (2017b)	Male/Female	College students	343	USA	Basketball, golf, ice hockey, soccer, tennis, football, softball, wrestling, volleyball	Retrospective	Talent
Post et al. (2017a)	Male/Female	Grades 9–12	1,544	USA	Gymnastics, ice hockey, lacrosse, soccer, swimming, tennis, track, volleyball, wrestling	Cross-sectional	Injury and sex
Rugg et al. (2018)	Male	Adults	237	USA	Basketball	Descriptive epidemiological study	Injury and performance
Russell (2014)	Male/Female	17–22	200	USA	Basketball, softball, soccer, football, baseball, volleyball, tennis, track cheer, gymnastics, dance, swimming, wrestling, badminton, bowling, boxing, hockey, mixed martial arts, tae kwan do	Retrospective	Physical activity and sport motivation
Russell and Limle (2013)	Male/Female	18–22	153	USA	Baseball, basketball, cheer, cross-country, football, gymnastics, ice hockey, soccer, softball, swimming, track, volleyball, wrestling, golf	Retrospective	Physical activity and sport motivation
Russell and Molina (2018)	Female	High school	77	USA	Soccer, volleyball, tennis	Cross-sectional	Motivation and burnout
Santos et al. (2015)	Male/Female	College students	34	Portugal	Basketball, football, rugby	Case control	Transfer
Santos et al. (2017)	Male/Female	College students	76	Portugal	Soccer, basketball, volleyball, indoor soccer, handball, rugby, roller hockey, swimming, table tennis, karate, athletics, trampoline, gymnastics, canoeing, kickboxing, tennis, dance, judo, pentathalon, badminton, equestrian, bodyboarding	Cross-sectional	Basketball skills
Storm et al. (2012)	Male/Female	18–40	17	Denmark	Swimming, sailing, orienteering, golf, gymnastics, handball, soccer, badminton, kayak, rowing	Retrospective	Talent and culture
Strachan et al. (2009)	Male/Female	12–16	74	Canada	Swimming, artistic gymnastics, rhythmic gymnastics, diving	Case control	Sport experiences, personal development, and sport outcomes, namely enjoyment and burnout
Swindell et al. (2019)		Over 18	303	USA	All NCAA division 1 sport	Cross-sectional	Motivation for specializing and age of specializing
Wilhelm et al. (2017)	Male	22–40	102	USA	Baseball	Descriptive epidemiological study	Injury and effectiveness
**Specialization General**							
Arede et al. (2019)	Both	Under 13	68	Portugal	Basketball	Retrospective	Talent
Baker et al. (2005)	Male	24–40	28	Canada	Triathlete	Retrospective	Talent
Coutinho et al. (2015)	Both	23 or older	60	Portugal	Volleyball	Retrospective	Talent
Cupples et al. (2018)	Male	18–34	224	Australia	Rugby	Retrospective	Talent
Fransen et al. (2012)	Male	6–12	735	Belgium	Did not specify	Cross-sectional	Physical fitness, motor coordination
Güllich (2014)	Male	Adults	54	Germany	Field hockey	Retrospective	Talent
Güllich (2017)	Both	25–30	166	Germany	All Olympic sports	Retrospective	Talent
Güllich and Emrich (2014)	Both	Olympic athletes	1,558	Germany	All Olympic sports	Retrospective/longitudinal	Talent
Güllich and Emrich (2013)	Both	Adults	695	Germany	All Olympic sports	Retrospective	Talent
Hendry and Hodges (2018)	Male	15–20	102	UK	Soccer	Retrospective	Talent
Leite and Sampaio (2012)	Male	7–35	1,170	Portugal	Basketball	Retrospective	Talent
Leite et al. (2009)	Male	Adults	112	Portugal	Roller-hockey, soccer, volleyball, basketball	Retrospective	Talent
Leite et al. (2013)	Male	Older than 24	120	Portugal	Basketball	Retrospective	Talent
Livingston et al. (2016)	Both	7–11	59	USA	Soccer	Cross-sectional	Parents perceptions and reasons for participating
Moesch et al. (2013)	Both	Adults	185	Denmark	Soccer, handball, ice hockey, volleyball	Retrospective	Talent
Moesch et al. (2011)	Both	Adults	243	Denmark	CGS sports	Retrospective	Talent
Sieghartsleitner et al. (2018)	Did not specify	u13–u18	294	Switzerland	Soccer	Retrospective	Talent
Sugimoto et al. (2019)	Female	12–18	236	USA	Did not specify	Cross-sectional	Injury
Wall and Côté (2007)	Male	13–15	12	Canada	Ice hockey	Retrospective	Dropout and investment
Zibung and Conzelmann (2013)	Male	Adults	159	Switzerland	Soccer	Retrospective	Talent

Finally, specialization general studies (i.e., did not explicitly include specialization in the purpose) were largely comprised of talent development studies (*n* = 16). These studies generally focused on the developmental activities of athletes who became elite or differences between elite and non-elite athletes, which meant that while early specialization was a focus in the article, the actual purpose of the paper was not necessarily to advance understanding of early specialization. The average sample size was 314 with a range from 12 to 1,558. Studies were retrospective (*n* = 17) or cross-sectional (*n* = 3) with one having a combined longitudinal/retrospective design. Participants were generally either males only or mixed samples of males and females, with only one study examining females only. Lastly, data was collected in nine different countries.

### Definitions and Measures

As the second objective of the review was to examine how early specialization has been defined and measured, this section focuses on the conceptual and operational definition of early specialization as well as the approaches used to determine early specializers, across all types of papers. In their 2019 scoping review, DiSanti and Erickson (2019) established that year-round intense training in a single sport at the exclusion of other sports was the most commonly used definition in empirical studies. In line with this review of empirical studies, the four key components of this definition were used as a starting point for our analysis (i.e., year-round, intense training, single sport, and exclusion of other sports). Deliberate practice was also added as a definition component, as the previously mentioned Developmental Model of Sport Participation (Côté and Fraser-Thomas, 2016) suggests deliberate practice is also a key indicator of early specialization. Finally, as this review focuses on early specialization definitions were also coded depending on whether they included any information regarding an age threshold.

Definitions were extracted from all 129 articles and coded for each of the six individual components (i.e., year-round, intense training, single sport, exclusion of other sports, deliberate practice, and age threshold) of early specialization, which are presented in Table 4. Just over 20% (i.e., 20.9%, *n* = 27) of the articles included the initial four-component definition of early specialization. The most frequent individual component of early specialization was single sport participation (i.e., 73.6%, *n* = 95), while the least frequent individual component was high amounts/volume of deliberate practice at 9.3% (*n* = 12). Additionally, 44.2% (*n* = 57) included year-round training, 41.9% (*n* = 54) used exclusion of other sports and 31.8% (*n* = 41) considered intense training to be a key facet of early specialization. A particularly interesting finding was the lack of distinction between early specialization vs. sport specialization; only 30.2% (*n* = 39) of the papers included some mention of early or young age as part of the definition for early specialization. Finally, 17.1% (*n* = 22) of the 129 papers discussed and focused on early specialization yet had no explicit definition of early specialization.

**Table 4:** Definitions provided for all studies.

**References**	**Definition provided**	**Year round**	**Intense training**	**Single sport**	**Exclusion of other sports**	**Young age**	**Deliberate practice**
American Academy of Pediatrics (2000)	None						
Anderson and Mayo (2015)	Exclusive participation in a single sport on a year-round basis, with a primary focus on training and development in that sport.	Yes		Yes	Yes	Yes	
Arede et al. (2019)	None						
Baker and Robertson-Wilson (2003)	Limiting sport participation to a single sport with the specific goal of guiding the child athlete to top achievement			Yes		Yes	
Baker et al. (2005)	A shift from activities that are play like in nature to more structured and effortful training activities. In addition, the number of sport-specific training hours dramatically increases from initial involvement in the sampling years to committed involvement in the investment years						
Baker et al. (2009)	Four specific parameters: early start age in sport; early involvement in one sport (as opposed to participating in several sports); early involvement in focused, high intensity training; and early involvement in competitive sport		Yes	Yes		Yes	
Baker (2003)	To limit their childhood sport participation to a single sport, with a deliberate focus on training and development in that sport			Yes		Yes	
Beese et al. (2015)	Year-round participation in a single sport to the exclusion of other sports and activities	Yes		Yes	Yes		
Bell et al. (2016)	Year-round intensive training in a single sport at the exclusion of other sports	Yes	Yes	Yes	Yes		
Bell et al. (2018c)	Participation in a single sport at the exclusion of other sports			Yes	Yes		
Bell et al. (2018b)	Year-round participation in sport at the exclusion of other sports	Yes		Yes	Yes		
Bell et al. (2018a)	Intense, year-round training in a single sport and may include the exclusion of other sports	Yes	Yes	Yes	Yes		
Bell (2018)	None						
Black et al. (2019)	Participation in ice hockey at the exclusion of other sports at or before the age of 12 years			Yes	Yes	Yes	
Blagrove et al. (2017)	Training routines that focus on intensive training in a single sport (for 0.8 mo/y), or a total weekly training volume which exceeds the athletes' age in years, until late adolescence		Yes	Yes			
Bodey et al. (2013)	Emphasizes focused training in a single sport on a year-round basis	Yes		Yes			
Branta (2010)	None						
Brenner (2016)	An athlete focuses on only 1 sport, usually at the exclusion of any other and often year-round	Yes		Yes	Yes		
Bridge and Toms (2013)	Continual year-round training and development in a single sport between the ages of 6 and 12 years	Yes		Yes		Yes	
Brooks et al. (2018)	Specialize in a single sport at the exclusion of other sports			Yes	Yes		
Brylinsky (2010)	None						
Buckley et al. (2017)	intense, year-round [8 months/year] training in a single sport with the exclusion of other sports	Yes	Yes	Yes	Yes		
Buhrow et al. (2017)	Engaging in year-round training in one regulated, competitive sport at the elimination of all other sports	Yes		Yes	Yes		
Callender (2010)	None						
Capranica and Millard-Stafford (2011)	As the age or point in time in an athlete's development when sports training and competition is restricted to and focused upon a single sport in the pursuit of elite performance			Yes			
Carson et al. (2010)	None						
Coakley (2010)	Year-round specialization in a single sport	Yes		Yes			
Côté and Hancock (2016)	None						
Côté et al. (2009)	Investing in one sport on a year round basis from a young age with the goal of developing expertise	Yes		Yes		Yes	
Côté et al. (2009)	A high volume of deliberate practice and a low amount of deliberate play in one sport and focuses on performance as early as age six or seven					Yes	Yes
Coutinho et al. (2015)	An early start age in doing one specific sport and an early investment in deliberate practice (i.e., highly structured and intensive activities, with the explicit goal of improving performance)		Yes	Yes		Yes	Yes
Cupples et al. (2018)	Single-sport involvement, low deliberate play and progressive investment in deliberate practice with age			Yes			Yes
DePhillipo et al. (2018)	Intense training year-round in a specific sport starting at a young age	Yes	Yes	Yes		Yes	
DiCesare et al. (2019)	A year- or near year-round commitment to one sport at the exclusion of others	Yes		Yes	Yes		
DiFiori et al. (2017)	None						
DiFiori et al. (2014)	Intensive, year-round training in a single sport at the exclusion of other sports	Yes	Yes	Yes	Yes		
DiSanti and Erickson (2019)	Intensive year-round training in a single sport at the exclusion of other sports	Yes	Yes	Yes	Yes		
DiStefano et al. (2018)	Only participate in 1 sport at an early age, with goals of achieving elite athletic success			Yes		Yes	
Fabricant et al. (2016)	Year-round intensive training in a single sport at the exclusion of other sports	Yes	Yes	Yes	Yes		
Feeley et al. (2016)	Intensive, year-round training in a single sport to the exclusion of other sports	Yes	Yes	Yes	Yes		
Ferguson and Stern (2014)	Intense year round training in a specific sport with the exclusion of other sports at a young age	Yes	Yes	Yes	Yes	Yes	
Ford et al. (2012)	Begin during childhood in relatively high intensity practice and competition in their primary sport. They engage in relatively little play activity in the primary sport and in relatively few or no other sports during this period. It usually also involves identification and selection into a talent development programme in the primary sport during childhood		Yes	Yes		Yes	
Fransen et al. (2012)	Enter their primary sport at an early age and participate in a high amount of deliberate practice in their primary sport with almost no deliberate play in any other sports					Yes	Yes
Gallant et al. (2017)	Early sport specializers (i.e., high OPA level, low UPA level, and participation in 1 sport only)			Yes			
Geisler (2019)	Three primary criteria: (1) intensive training or competition in organized sports for more than 8 months per year, (2) participating in one sport to the exclusion of participation in others, and (3) involving pre-pubertal aged children prior to 12 years		Yes	Yes	Yes	Yes	
Ginsburg et al. (2014)	Which necessitates a high volume of deliberate practice in a single sport as early as six or seven years of age and a purposeful focus on training and skill development			Yes		Yes	Yes
Gonçalves et al. (2012)	None						
Goodway and Robinson (2015)	Focused involvement in one sport and a large number of hours of deliberate practice with the goal of improving sport skills and performance outcomes during childhood			Yes		Yes	Yes
Gould (2010)	None						
Griffin (2008)	None						
Güllich (2014)	Reinforced intensity and expansion of domain-specific practice,		Yes				
Güllich (2017)	Early concentration in one sport with reinforced sport specific DP/training that is subsequently expanded through all age periods			Yes		Yes	Yes
Güllich and Emrich (2014)	None						
Güllich and Emrich (2013)	Represent the poles of a continuum differing in exclusivity and intensity of early, sport-specific practice/training, involvement in different sports and non-organized sport activities.		Yes	Yes	Yes		
Hall et al. (2015)	With intense year-round training in a single sport at the exclusion of other sports or activities	Yes	Yes	Yes	Yes		
Hastie (2015)	Intense, year-round training in a single sport with the exclusion of other sports	Yes	Yes	Yes	Yes		
Haugaasen and Jordet (2012)	High amount of deliberate practice, low deliberate play, one sport			Yes			Yes
Huxley et al. (2017)	High volumes of domain specific deliberate practice in one sport from an early age			Yes		Yes	Yes
Hill (1993)	Limited their participation to one sport which they practiced trained and competed in year round	Yes		Yes			
Horn (2015)	None						
Jayanthi et al. (2015)	Year-round intensive training in a single sport at the exclusion of other sports	Yes	Yes	Yes	Yes		
Jayanthi and Dugas (2017)	Year-round intense training in a single sport with the exclusion of other sports	Yes	Yes	Yes	Yes		
Jayanthi et al. (2013)	Intense, year-round training in a single sport with the exclusion of other sports	Yes	Yes	Yes	Yes		
Jayanthi et al. (2018)	Intensive, year-round training in a single sport at the exclusion of other sports	Yes	Yes	Yes	Yes		
Kaleth and Mikesky (2010)	This practice typically involves children (ages 6 to 12) who commit almost exclusively to a single sport, train and compete year-round, and have high internal—and often external—expectations	Yes		Yes		Yes	
Landers et al. (2010)	Specialization in one sport or in one position in a sport, at increasingly younger ages, in order to compete at the highest levels			Yes		Yes	
LaPrade et al. (2016)	1. Participation in intensive training and/or competition in organized sports > 8 months per year (essentially year round) 34 2. Participation in 1 sport to the exclusion of participation in other sports (limited free play overall) 33 3. Involving pre-pubertal (seventh grade or roughly age 12 years) children.	Yes	Yes	Yes	Yes	Yes	
Larson et al. (2019)	(a) involving pre-pubertal children; who (b) participate in one sport to the exclusion of others, with limited free play overall; and (c) participate in intensive training and/or competition in organized sports for more than 8 months/year		Yes	Yes	Yes	Yes	
Leite and Sampaio (2012)	Involved targeted involvement in a single sport			Yes			
Leite et al. (2009)	A shift from activities that are play-like in nature to more structured and effortful training activities. In addition, the number of sport-specific training hours dramatically increases from initial involvement in the sampling years to committed involvement in the investment years						
Leite et al. (2013)	Limit their childhood sport participation to a single sport, with a deliberate focus on training and development in that sport			Yes			
Livingston et al. (2016)	Deliberate practice or training with the purpose of improving skills						Yes
Malina (2010)	Specialized, systematic training in a single sport at a relatively young age with the goal of attaining elite status			Yes		Yes	
Martin et al. (2017)	Participate in a single sport on a year-round basis, with a focus on training and development in that single sport	Yes		Yes			
Mattson and Richards (2010)	Characterized by participation in specific, intense training for a single sport at a competitive level at an early age		Yes	Yes		Yes	
Matzkin and Garvey (2019)	Intensive year-round training in a single sport at the exclusion of other sports	Yes	Yes	Yes	Yes		
McDonald et al. (2019)	Intensive training/competition in organized sports >8 months per year, participation in one sport with the exclusion of all other sports, and involvement of children who are pre-pubertal or ~12 years of age		Yes	Yes	Yes	Yes	
McFadden et al. (2016)	Specialize in one sport before the age of 12, youth engage in high amounts of deliberate practice in a single sport			Yes		Yes	Yes
McGuine et al. (2017)	None						
McLeod et al. (2019)	Extensive year-round training in a single sport at the exclusion of others	Yes		Yes	Yes		
Mendes et al. (2018)	Young children may have an early starting age in highly structured and intensive activities with the explicit goal of improving performance in a sport		Yes				
Miller et al. (2017)	Year-round intensive training in a single sport at the exclusion of other sports	Yes	Yes	Yes	Yes		
Moesch et al. (2013)	Early involvement in the main sport, often occurring in early to middle childhood, with very little or no involvement in other sports			Yes	Yes	Yes	
Moesch et al. (2011)	Normally occurring in early to middle childhood, with little or no involvement in other sports. Additionally, the importance of a high amount of deliberate practice is stressed during all ages			Yes	Yes	Yes	Yes
Moseid et al. (2019)	The time when the athlete defined one sport as being more important than other sports						
Mostafavifar et al. (2013)	Year-round sport-specific training, participation on multiple teams of the same sport and focused participation in a single sport	Yes		Yes			
Myer et al. (2015)	Intensive year-round training in a single sport at the exclusion of other sports	Yes	Yes	Yes	Yes		
Myer et al. (2016)	Including year-round sport-specific training, participation on multiple teams of the same sport, and focused participation in a single sport	Yes		Yes			
NASPE Staff (2006)	None						
Noble and Chapman (2018)	None						
Normand et al. (2017)	Intense, year-round training program in a single sport at the exclusion of other activities	Yes	Yes	Yes	Yes		
Padaki et al. (2017a)	Focusing on a sport to the exclusion of other sports and playing and training in the sport more than 8 months per year prior to the age of 12			Yes	Yes	Yes	
Padaki et al. (2017b)	The combination of playing and training in a single sport for > 8 months per year, playing a single sport “to the exclusion of participation in other sports,” and starting this commitment prior to age 12 years			Yes	Yes	Yes	
Pantuosco-Hensch (2006)	Athletes limiting their participation to one sport which is practiced, trained for and/or competed in on a year round basis	Yes		Yes			
Pantuosco-Hensch (2010)	Athletes limit their athletic participation to one sport which is practiced, trained for, and competed in throughout the year	Yes		Yes			
Pasulka et al. (2017)	Year-long, intensive training in a single sport at the exclusion of other sports	Yes	Yes	Yes	Yes		
Patel and Jayanthi (2018)	Intense, year-round training in a single sport with exclusion of other sports	Yes	Yes	Yes	Yes		
Post et al. (2017c)	Year-round intensive training in a single sport at the exclusion of other sports	Yes	Yes	Yes	Yes		
Post et al. (2017b)	Year-round participation in a single sport at the exclusion of other sports	Yes		Yes	Yes		
Post et al. (2017a)	Year-round, intensive training in a single sport at the exclusion of other sports	Yes	Yes	Yes	Yes		
Read et al. (2016)	As early age involvement in one chosen sport during the period of early-to middle childhood (up to age 13 years) with no subsequent participation in the other sports or activities available			Yes	Yes	Yes	
Reider (2017)	Specializing in one sport to the exclusion of all others			Yes	Yes		
Rugg et al. (2018)	Intensive year-round training in a single sport at the exclusion of other sports	Yes	Yes	Yes	Yes		
Russell (2014)	Limited their participation to one sport which they practiced trained and competed in year round	Yes		Yes			
Russell and Limle (2013)	Limited their participation to one sport which they practiced trained and competed in year-round	Yes		Yes			
Russell and Molina (2018)	Limited their participation to one sport, which was practices for and competed in throughout the year, to the exclusion of other activities	Yes		Yes	Yes		
Santos et al. (2015)	These categories follows the guidelines of the Long-Term Athlete Development model						
Santos et al. (2017)	Specialized participation in early childhood, promoting highly structured training as the answer to current competitive demands					Yes	
Sieghartsleitner et al. (2018)	Whether young talents should focus on a single sport specific domain early			Yes		Yes	
Sluder et al. (2017)	An athlete participating in a single main sport on a year-round basis (>8 months per year) and/or quitting all other sports to focus on a single sport	Yes		Yes	Yes		
Smith (2015)	Limiting participation to one sport which is practiced, trained for and/or competed in on a year-round basis	Yes		Yes			
Smith et al. (2017)	None						
Smucny et al. (2015)	Intensive, year-round training in a single sport at the exclusion of other sports	Yes	Yes	Yes	Yes		
Stewart and Shroyer (2015)	None						
Storm et al. (2012)	None						
Strachan et al. (2009)	Investing at least 15 h per week in their respective sports and involved from a young age					Yes	
Sugimoto et al. (2019)	Year-around (more than 8 months per year) and quitting other sports in order to focus on one sport	Yes		Yes	Yes		
Sugimoto et al. (2017)	Year-round, high-intensity training specialized to a single sport at an early age	Yes	Yes	Yes		Yes	
Swindell et al. (2019)	Year-round training and participation in a single sport at the exclusion of other sports	Yes		Yes	Yes		
Torres (2015)	A practice in which young athletes commit to train and compete almost exclusively in a single sport			Yes		Yes	
Waldron et al. (2019)	High intensity, year-round training in a single sport, with the exclusion of other sports	Yes	Yes	Yes	Yes		
Wall and Côté (2007)	Intense training in one sport at a young age		Yes	Yes		Yes	
Walters et al. (2018)	None						
Weiss (2015)	None						
Wiersma (2000)	Year-round training in a single sport at the exclusion of other sport or non-sport activities	Yes		Yes	Yes		
Wilhelm et al. (2017)	Intense, year-round training in a single sport with the exclusion of other sports	Yes	Yes	Yes	Yes		
Williams (2018)	Participation in a single sport and reporting more than 8 months per year training for that sport			Yes			
Wilson (2006)	Year-round training in a single sport at the exclusion of other activities	Yes		Yes	Yes		
Zibung and Conzelmann (2013)	None			Yes		Yes	

While definitions lay the foundation for understanding components of early specialization, it follows that studies in turn must classify athletes according to these definitions. Further analysis was conducted on the measures used in the 48 data-driven specialization specific studies in order to better understand how researchers classified athletes as early specializers. A key step to measuring early specialization is determining what is meant by early, yet only 25 studies (52.1%) included a measure of age in their screening tool. Of those, 56% (*n* = 14) used “before the age of 12” as the cut-off for early specialization. To determine specialization status in the empirical studies, 18 different approaches or strategies were employed. It should be noted that while different indicators of early specialization were used, some of the constructs overlap. Fifteen (31.3%) of the 48 studies used the “Sport Specialization Scale” by Jayanthi et al. (2015), 11 (22.9%) used a single item question (e.g., “Did you specialize before high school, yes or no?”) while 10 (20.8%) collected a full developmental history of the athlete (e.g., hours in each sport, practice history, and number of sports at different ages). For a complete list of the different approaches used, see Table 5.

**Table 5:** Measures used to determine early specialization.

**References**	**Specialization determined by**	**Young age measured**	**Age used for young**	**Measure of intensity**
Beese et al. (2015)	Single vs. multi-sport	No	None	No
Bell et al. (2016)	Sport Specialization Scale/ Single vs. multi-sport	No	None	No
Bell et al. (2018b)	Sport Specialization Scale	No	None	No
Bell et al. (2018a)	Sport Specialization Scale	No	None	No
Black et al. (2019)	Full history	Yes	Before 12	No
Bridge and Toms (2013)	Single vs. multi-sport	Yes	6–12	No
Brooks et al. (2018)	Sport Specialization Scale	No	None	No
Buckley et al. (2017)	Single Item “Did you quit other sports to focus on one sport?”	No	None	No
Buhrow et al. (2017)	Single item “At what age did you specialize in year-round training in one sport?”	Yes	14	No
DePhillipo et al. (2018)	Case report	Yes	11	Yes
DiCesare et al. (2019)	Single vs. multi-sport	No	None	No
DiStefano et al. (2018)	Sport Specialization Scale	Yes	Did not Specify	No
Ferguson and Stern (2014)	Case report	No	None	Yes
Ford et al. (2012)	Full history	Yes	6–12	No
Gallant et al. (2017)	Full history	Yes	6–11	No
Ginsburg et al. (2014)	Full History	Yes	Before 12	No
Hall et al. (2015)	Full history	No	None	No
Hill (1993)	Single Item “did you specialize during highschool?”	No	None	No
Ferguson and Stern (2014)	Sport Specialization Scale	Yes	Did not specify	No
Jayanthi et al. (2018)	Sport Specialization Scale	No	None	No
Larson et al. (2019)	Full History	Yes	Before 12	Yes
Martin et al. (2017)	Single item “Did you specialize before college?”	Yes	Did not specify	No
McDonald et al. (2019)	Single item “What age did you specialize?”	Yes	Before 12	No
McFadden et al. (2016)	Sport Specialization Scale	Yes	Before 12	No
McGuine et al. (2017)	Sport specialization Scale	No	None	No
McLeod et al. (2019)	Sport Specialization Scale	No	None	No
Mendes et al. (2018)	Full history	Yes	Before 12	No
Miller et al. (2017)	Sport Specialization Scale	No	None	No
Moseid et al. (2019)	Single item “At what age did you decide to focus on your sport?”	Yes	Before 12	No
Noble and Chapman (2018)	Did not specify	Yes (sport specific)	19–23	No
Padaki et al. (2017a)	3 item importance scale	Yes	Did not specify	No
Padaki et al. (2017b)	Self-assignment	No	None	No
Pantuosco-Hensch (2010)	Full history	Yes	Before 12	No
Pasulka et al. (2017)	Sport Specialization Scale	No	None	No
Patel and Jayanthi (2018)	Qualitative interview	No	None	No
Post et al. (2017c)	Sport Specialization Scale	No	None	No
Post et al. (2017b)	Sport Specialization Scale	No	None	No
Post et al. (2017a)	Sport specialization Scale	No	None	No
Rugg et al. (2018)	Single vs. multi sport	No	None	No
Russell (2014)	Single item “did you specialize as a youth?”	Yes	Before Adolescence	No
Russell and Limle (2013)	Single item “did you specialize as a youth?”	Yes	Before 15	No
Russell and Molina (2018)	Single item “Are you a specializer or not?”	No	None	No
Santos et al. (2015)	Full History	Yes	Did not specify	No
Santos et al. (2017)	Full history	Yes	Before 12	No
Storm et al. (2012)	Qualitative Interview	Yes	Before 12	No
Strachan et al. (2009)	Hours per week	No	None	No
Swindell et al. (2019)	Did not specify	Yes	Before 12	Did not specify
Wilhelm et al. (2017)	Single item “Did you specialize before high school, yes or no?”	Yes	Before high school	No

## Discussion

Early specialization is currently a “hot button” topic in athlete development research in particular and sport science more generally. Our review suggests much of the discussion in this area is driven by non-data driven, commentaries, editorials, and reviews, which undermines the extent to which recommendations about early specialization can be seen as evidence-based. Only 37% of the literature in this review included data-driven studies that were explicitly designed to advance our understanding of early specialization specifically, with 43% of the papers comprised of editorials, commentaries, or reviews. Common rhetoric around this issue assumes early specialization leads to injury, yet only 14 studies have actually examined this relationship with certain indicators of early specialization and of those only five measured early specialization. Similarly, despite broad recommendations that early specialization increases risk of burnout from sport, only three studies explicitly examined this relationship. Given the findings contained in this review, we believe there is insufficient evidence to provide the foundation for the strong and “conclusive” position statements around this topic. Importantly, there is also insufficient evidence to conclude there are no risks to early specialization. Despite messages to the contrary, the benefits and risks of early specialization remains an open topic for sport researchers.

The work summarized in this review raises important concerns about the state of the evidence against early specialization and how future research could be improved to resolve outstanding issues. The first issue relates to the conflating of “early specialization” and “sport specialization.” Most researchers would agree that the considerable training required to become an elite athlete necessitates eventual specialization at some point (Baker et al., 2003). Researchers however advised against the practice of early specialization, suggesting this leads to negative outcomes such as increased injury rates (Hall et al., 2015) without associated benefits (Baker, 2003). In the current review, only half of the studies identified measured an aspect of “early.” This distinction between “early specialization” and “specialization” is important. “Specialization” in a single sport may be associated with injury or other negative outcomes due to the link between specialization and overtraining (Ferguson and Stern, 2014) not the age at which it is occurring. Further, in order to properly study the effects of early specialization, it is important to clearly operationalize “early.” Of the few studies in this review that measured early only about half used the same criteria (i.e., before age 12).

Another issue relates to the validity of the scales or tools used to determine specialization. The most commonly used scale is Jayanthi et al.'s (2015) Sport Specialization Scale, which uses three criteria [1. single sport training, 2. exclusion of other sports, and 3. year-round training (>8 months)] to rank athletes as low (having only one of the criteria), moderate (two of three) or high on specialization (all three). Over 30% of the data- driven specialization specific studies in this review used this scale, despite concerns about the validity of this scale (Smith et al., 2017). With this scale, for example, a recreational athlete who participates once a week for 2 h in basketball, but quit soccer at age seven, would be regarded as more specialized than a competitive basketball player who participates for 6 h a week but only ever participated in basketball, despite the fact that most practitioners would be more concerned about the latter. Furthermore, 20% of studies in this review used only a single item to measure specialization, raising further concerns about whether a single item is nuanced enough to adequately capture this multi-faceted concept. As noted in the results, 18 different approaches have been used to determine specialization status often inconsistently categorizing athletes. For instance, one study compared a self-classification method (i.e., are you a single sport or multi-sport athlete) to the 3-point “Sport Specialization Scale,” resulting in only 38% agreement on the athletes' categorization and differing results on the relationships between specialization status and injury history (Bell et al., 2016).

There were also inconsistencies between the definitions of early specialization and the markers researchers used to measure it. Over half of the studies mentioned “intense” training in their definition of early specialization yet only three studies included any measure of intensity. These were unique case reports that collected a thorough background on one athlete. This misalignment between definition and method further highlights concerns with validity that mar this area of research.

These issues highlight the precarious foundation of the early specialization evidence base. Ferguson and Stern (2014) noted “All position statements are slightly different, but there is not one single position statement that supports early specialization” (p. 380)—but it is unclear why researchers have been so quick to conclude against early specialization given the lack of a consistent definition or method of classifying athletes. Also concerning is that researchers are recommending multi-sport participation in lieu of early specialization (Coté et al., 2009) without understanding the harmful mechanism behind early specialization.

Around 73% of the papers in this review agreed that single-sport participation was a key component of early specialization, yet this component of specialization alone was not found to be associated with injury history (Bell et al., 2016). The harmful mechanisms behind early specialization are undoubtedly more complex than just single-sport participation and advocating for multi-sport participation without fully understanding what aspect of early specialization is harmful may be short-sighted.

### Future Directions

There are several important next steps for research in this area. First and most important, there needs to be a clear and consistent definition of early specialization that can be utilized across disciplines, organizations and researchers. The field will be unable to understand the potential consequences or benefits of early specialization without first establishing a clear understanding of the components and the requirements of this concept. Although it may be difficult to come to a consensus on a definition for early specialization, a Delphi-type approach (i.e., using experts' answers to questionnaires) could be a useful way to reach convergence. Experts could reflect on which previously used facets of early specialization are essential, which are less important, and which are missing. This could help the field establish a definition of early specialization that most agree with. Second, a valid and reliable scale that captures and categorizes early specializers is needed. Any future scales should include some measure of age in order to distinguish “early specialization” from “sport specialization.” Additionally, researchers may consider adding measures of intensity to the classification of early specializers to separate those who participate more recreationally from those at risk of overtraining. As noted by a previous systematic review (DiSanti and Erickson, 2019), 92.5% of studies used a dichotomy (i.e., specializer or not) to classify athletes. This likely over-simplifies a highly nuanced topic and future research should consider establishing a continuum of early specialization. Finally, there is a need for more research overall on this topic. Suggestions and statements need to be evidence-based and in order for that to happen there needs to be more evidence.

### Limitations

While this review provides the first comprehensive look at all papers related to early specialization in sport, it is not without limitations. First the inclusion of non-data driven studies, while important for understanding the composition of the literature, made it impossible to synthesize all papers in the review uniformly. Additionally, the range of approaches used to classify athletes also made it impractical to perform a meta-analysis. Second, the inclusion criteria that studies “explicitly use the term ‘specialization”’ might have eliminated studies that focused on the same area but used other words to describe this pattern of participation. Finally, while the search strategy was created in consultation with a profession research librarian, the search string used could have limited the number of studies found through each of the four search engines (e.g., using the connector “AND” could have excluded studies that did not include all the required search terms but were still relevant to the review).

## Conclusion

This review has shown that there are troubling inconsistencies in the definitions of early specialization and the approaches used to classify athletes. Although this review does not directly establish a clear and consistent definition of early specialization, it is an essential first step. While practitioners and organizations advise against early specialization, this review raises significant questions around the validity and reliability of the evidence underpinning these claims. Once a consistent definition of early specialization is established and researchers have created a valid and reliable measure to capture it, the work to determine negative consequences and benefits of early specialization can begin. Until then research and any recommendations around early specialization should be viewed with caution. To understand the mechanisms behind early specialization and *why* it is potentially harmful or beneficial, the field must first establish *what* early specialization is and *how* best to measure it.

## Data Availability Statement

The raw data supporting the conclusions of this article will be made available by the authors, without undue reservation.

## Author Contributions

AM collected the data and drafted the manuscript. All authors read, edited, and approved the final manuscript and designed the study.

## Conflict of Interest

The authors declare that the research was conducted in the absence of any commercial or financial relationships that could be construed as a potential conflict of interest.

## Footnotes

^1^Book chapters were not included in the review as they do not undergo a rigorous peer-review process and they are not indexed the way journals are, therefore they would not show up in our four databases.
